# Supplementary material for: Association between well-being and compliance with COVID-19 preventive measures by healthcare professionals: A cross-sectional study
Source: PLoS One. 2021 Jun 7;16(6):e0252835. doi: 10.1371/journal.pone.0252835 (PMC8183980; doi:10.1371/journal.pone.0252835)
Supplement: S1 File — (DOCX) [file pone.0252835.s001.docx]

**S1 File**

**S1 Table. Survey Distributed to the Respondents**

| **Knowledge** |
| --- |
| **Personal Hygiene** |
| COVID-19 CANNOT be transmitted by – mosquito bites vs. Door hands and hand-phone surfaces, sneezing and rubbing of eyes, not sure |
| Which medium can kill COVID-19 –soap and alcohol disinfectant vs. hot water, hand dryers, not sure |
| **Social Distancing** |
| How far apart should people stand or sit - >1 or 2m vs. not equal to >1 or 2 m |
| **Attitude** |
| **Personal Hygiene** |
| I am interested in increasing my knowledge about hygiene measures |
| Wearing a facemask is important during COVID-19 Pandemic – to protect self and others vs. don’t think it is important, government ordered, family members asked to wear |
| **Social Distancing** |
| Do you think that social distancing measures is important and will help to reduce the spread of - COVID-19 |
| I should stay at home when I am not feeling well |
| Which of the following would you consider as main reason for compliance with social distancing measures - Fear of self or family members getting COVID 19, Fear of fines/ punitive measures |
| Would you willingly participate in the contact tracing app |
| For how long are you willing to practice social distancing behaviour to keep yourself and others safe – as long as it takes, limited time (not more than 6 months) |
| **Practices** |
| **Personal Hygiene** |
| *How often do you wash your hands with soap or alcohol- based disinfectant a day |
| *Do you wash your hands before and after handing food |
| *Do you cover your mouth when you sneeze or cough |
| *Do you wear a mask in public |
| Do you usually wear a mask when you have flu-like symptoms before the COVID-19 pandemic |
| *Do you AVOID touching your eyes nose and mouth during COVID-19 pandemic |
| *Do you avoid shaking hands |
| *Do you wipe surfaces and objects with disinfectant regularly |
| Do you watch out for surfaces that could be contaminated |
| **Social Distancing** |
| *Do you avoid standing or sitting close to people |
| How often do you go out of the house in a week (excluding going out for work |
| How many people do you meet face-to-face (<1m) apart everyday (excluding own household) |
| On average, how many places do you go in a day (excluding home) |
| **Source of information** |
| What is your preferred source of obtaining information with regards to COVID 19 |
| **Effect of social distancing on well-being (past month)** |
| What do you think your probability of getting COVID19 is in the next 1 month – 0%, < 50% and ≥ 50% |
| ***Emotional well-being*** |
| How often did you feel happy |
| How often did you feel interested in life |
| How often did you feel satisfied with life |
| ***Social well-being*** |
| How often did you feel that you had something to contribute to society |
| How often did you feel that you belonged to a community (like a social group, or your neighbourhood |
| How often did you feel that our society is a good place, or is becoming a better place for all people |
| How often did you feel that people are basically good |
| How often did you feel that the way our society works makes sense to you |
| ***Psychological well-being*** |
| How often did you feel that you liked most parts of your personality |
| How often did you feel that you are good at managing the responsibility of your daily life |
| How often did you feel that you had warm and trusting relationships with others |
| How often did you feel that you had experiences that challenged you to grow and become a better person |
| How often did you feel confident to think or express your own ideas and opinions |
| How often did you feel that your life has a sense of direction or meaning to it |

***Used to determine compliance amongst respondents in health services**

**Flourishing was defined as hedonic well-being symptoms (emotional well-being) ≥1 and positive functioning symptoms (social & psychological well-being) ≥6**

**S2 Table. KAP and mental health differences between HCP and non-HCP**

|  | **HCP (n=1,096) n (%)** | **Non-HCP (n=1,607) n (%)** | **p-value** |
| --- | --- | --- | --- |
| **Knowledge** | | | |
| **Personal Hygiene** |  |  |  |
| COVID-19 CANNOT be transmitted by |  |  | **<0.001** |
| Door hands and hand-phone surfaces | 10 (0.9) | 27 (1.7) |  |
| Mosquito bites | 987 (90.1) | 1347 (83.8) |  |
| Sneezing and rubbing of eyes | 48 (4.4) | 70 (4.4) |  |
| Not sure | 51 (4.7) | 163 (10.1) |  |
| Which medium can kill COVID-19? |  |  | **0.040** |
| Hand dryers | 9 (0.8) | 13 (0.8) |  |
| Hot water | 3 (0.3) | 18 (1.1) |  |
| Soap and alcohol disinfectant | 1072 (97.8) | 1547 (96.3) |  |
| Not sure | 12 (1.1) | 29 (1.8) |  |
| **Personal Hygiene Knowledge Score (mean, SD)** | 1.88 (0.35) | 1.80 (0.44) | **<0.001** |
| **Social Distancing** |  |  |  |
| How far apart should people stand or sit? |  |  | **<0.001** |
| >0.5m | 7 (0.6) | 22 (1.4) |  |
| >1 or 2m | 1057 (96.4) | 1529 (95.1) |  |
| >3 or 4m | 32 (2.9) | 56 (3.5) |  |
| **Attitude** | | | |
| **Personal Hygiene** |  |  |  |
| I am interested in increasing my knowledge about hygiene measures |  |  | 0.823 |
| Yes | 942 (85.9) | 1375 (85.6) |  |
| No | 154 (14.1) | 232 (14.4) |  |
| Wearing a facemask is important during COVID-19 Pandemic |  |  | **0.027** |
| I DO NOT think that wearing a facemask is important | 8 (0.7) | 20 (1.2) |  |
| Because government ordered me to wear a facemask | 19 (1.7) | 32 (2.0) |  |
| Because we can protect our self and others from COVID-19 | 1068 (97.4) | 1541 (95.9) |  |
| Because my family members asked me to wear a facemask | 1 (0.1) | 14 (0.9) |  |
| **Personal Hygiene Attitude Score (mean, SD)** | 1.67 (0.64) | 1.55 (0.74) | **<0.001** |
| **Social Distancing** |  |  |  |
| Do you think that social distancing measures is important and will help to reduce the spread of COVID-19? |  |  | 0.395 |
| Yes | 1076 (98.2) | 1581 (98.4) |  |
| No | 20 (1.8) | 26 (1.6) |  |
| I should stay at home when I am not feeling well |  |  | 0.388 |
| Yes | 1080 (98.5) | 1580 (98.3) |  |
| No | 16 (1.5) | 27 (1.7) |  |
| Which of the following would you consider as main reason for compliance with social distancing measures? |  |  | 0.243 |
| Fear of getting COVID 19 | 585 (53.4) | 839 (52.2) |  |
| Fear of family members getting COVID 19 | 455 (41.5) | 704 (43.8) |  |
| Fear of fines/punitive measures | 56 (5.1) | 64 (4.0) |  |
| Would you willingly participate in the contact tracing app? |  |  | **<0.01** |
| Yes | 883 (80.6) | 1187 (73.9) |  |
| No | 213 (19.4) | 420 (26.1) |  |
| For how long are you willing to practice social distancing behaviour to keep yourself and others safe? |  |  | 0.055 |
| As long as it takes | 739 (67.4) | 1135 (70.6) |  |
| For another 2-3 weeks | 47 (4.3) | 83 (5.2) |  |
| For another 1 month | 81 (7.4) | 118 (7.3) |  |
| For another 3 months | 113 (10.3) | 116 (7.2) |  |
| For another 6 months | 100 (9.1) | 127 (7.9) |  |
| I want social distancing to stop now | 16 (1.5) | 28 (1.7) |  |
| **Social Distancing Attitude Score (mean, SD)** | 3.50 (0.71) | 3.45 (0.70) | 0.094 |
| **Practices** | | | |
| **Personal Hygiene** |  |  |  |
| How often do you wash your hands with soap or alcohol- based disinfectant a day? |  |  | **<0.001** |
| High compliance | 891 (81.3) | 1095 (68.1) |  |
| Low compliance | 205 (18.7) | 512 (31.9) |  |
| Do you wash your hands before and after handing food? |  |  | 0.117 |
| High compliance | 1017 (92.8) | 1464 (91.1) |  |
| Low compliance | 79 (7.2) | 143 (8.9) |  |
| Do you cover your mouth when you sneeze or cough? |  |  | **0.006** |
| High compliance | 1052 (96.0) | 1503 (93.5) |  |
| Low compliance | 44 (4.0) | 104 (6.5) |  |
| Do you wear a mask in public? |  |  | 0.125 |
| High compliance | 1013 (92.4) | 1457 (90.7) |  |
| Low compliance | 83 (7.6) | 150 (9.3) |  |
| Do you usually wear a mask when you have flu-like symptoms before the COVID-19 pandemic? |  |  | **0.050** |
| High compliance | 461 (42.1) | 615 (38.3) |  |
| Low compliance | 635 (57.9) | 992 (61.7) |  |
| Do you AVOID touching your eyes nose and mouth during COVID-19 pandemic? |  |  | **<0.001** |
| High compliance | 853 (77.8) | 1118 (69.6) |  |
| Low compliance | 243 (22.2) | 489 (30.4) |  |
| Do you avoid shaking hands? |  |  | 0.274 |
| High compliance | 960 (87.6) | 1384 (86.1) |  |
| Low compliance | 136 (12.4) | 223 (13.9) |  |
| Do you wipe surfaces and objects with disinfectant regularly? |  |  | **<0.001** |
| High compliance | 667 (60.9) | 763 (47.5) |  |
| Low compliance | 844 (52.5) | 429 (39.1) |  |
| **Personal Hygiene Practice Score (mean, SD)** | 33.4 (4.58) | 31.8 (4.96) | **<0.001** |
| **Social Distancing** |  |  |  |
| Do you avoid standing or sitting close to people? |  |  | 0.353 |
| High compliance | 866 (79.0) | 1294 (80.5) |  |
| Low compliance | 230 (21.0) | 313 (19.5) |  |
| How often do you go out of the house in a week (excluding going out for work)? |  |  | **<0.007** |
| Never | 96 (8.8) | 171 (10.6) |  |
| 1-2 times | 595 (54.3) | 792 (49.3) |  |
| 3-4 times | 198 (18.1) | 322 (20.0) |  |
| 5-6 times | 123 (11.2) | 154 (9.6) |  |
| More than 7 times | 84 (7.7) | 168 (10.5) |  |
| How many people do you meet face-to-face (<1m) apart everyday (excluding own household)? |  |  | **<0.001** |
| 0 | 166 (15.1) | 480 (29.9) |  |
| 1-5 | 379 (34.6) | 731 (45.5) |  |
| 6-10 | 167 (15.2) | 198 (12.3) |  |
| 11-20 | 120 (10.9) | 78 (4.9) |  |
| >20 | 264 (24.1) | 120 (7.5) |  |
| On average, how many places do you go in a day (excluding home)? |  |  | **<0.001** |
| 0 | 128 (11.7) | 427 (26.6) |  |
| 1-2 | 828 (75.5) | 989 (1.5) |  |
| 3-4 | 110 (10.0) | 152 (9.5) |  |
| >4 | 30 (2.7) | 39 (2.4) |  |
| **Social Distancing Practice Score (mean, SD)** | 14.5 (2.64) | 15.5 (2.78) | **<0.001** |
| Compliance |  |  | **<0.001** |
| Low | 823 (75.1) | 1359 (84.6) |  |
| High | 273 (24.9) | 248 (15.4) |  |
| **Mental Health** | | | |
| What do you think your probability of getting COVID-19 is in the next 1 month? |  |  | **<0.001** |
| 0%, I will not get infected by COVID-19 | 273 (24.9) | 533 (33.2) |  |
| <25% | 555 (50.6) | 823 (51.2) |  |
| <50% | 202 (18.4) | 209 (13.0) |  |
| <75% | 59 (5.4) | 33 (2.1) |  |
| 100% | 7 (0.6) | 9 (0.6) |  |
| Effects of social distancing on mental health |  |  | **<0.001** |
| Not flourishing | 276 (25.2) | 504 (31.4) |  |
| Flourishing | 820 (74.8) | 1103 (68.6) |  |
| Total well-being |  |  | **0.009** |
| High | 419 (38.2) | 542 (33.7) |  |
| Low | 677 (61.8) | 1065 (66.3) |  |
| Emotional well-being |  |  | 0.081 |
| High | 530 (48.4) | 732 (45.6) |  |
| Low | 566 (51.6) | 875 (54.4) |  |
| Social well-being |  |  | **<0.001** |
| High | 398 (36.3) | 481 (29.9) |  |
| Low | 698 (63.7) | 1126 (70.1) |  |
| Psychological well-being |  |  | **0.042** |
| High | 498 (45.4) | 675 (42.0) |  |
| Low | 598 (54.6) | 932 (58.0) |  |
| **Total well-being Score (mean, SD)** | 46.2 (14.5) | 43.9 (15.0) | **<0.001** |

**S3 Table. KAP and mental health differences between HCP with low and high compliance**

|  | **Low compliance (N=823) n (%)** | **High compliance (N=273) n (%)** | **p-value** |
| --- | --- | --- | --- |
| **Knowledge** | | | |
| **Personal Hygiene** |  |  | 0.229 |
| COVID-19 CANNOT be transmitted by |  |  |  |
| Door hands and handphone surfaces | 7 (0.9) | 3 (1.1) |  |
| Mosquito bites | 750 (91.1) | 237 (86.8) |  |
| Sneezing and rubbing of eyes | 32 (3.9) | 16 (5.9) |  |
| Not sure | 34 (4.1) | 17 (6.2) |  |
| Which medium can kill COVID-19? |  |  | 0.985 |
| Hand dryers | 7 (0.9) | 2 (0.7) |  |
| Hot water | 2 (0.2) | 1 (0.4) |  |
| Soap and alcohol disinfectant | 805 (97.8) | 267 (97.8) |  |
| Not sure | 9 (1.1) | 3 (1.1) |  |
| **Personal Hygiene Knowledge Score (mean, SD)** | 1.89 (0.34) | 1.85 (0.40) | 0.080 |
| **Social Distancing** |  |  |  |
| How far apart should people stand or sit? |  |  | 0.268 |
| >0.5m | 6 (0.7) | 1 (0.4) |  |
| >1 or 2m | 797 (96.9) | 260 (95.2) |  |
| >3 or 4m | 20 (2.5) | 12 (4.4) |  |
| **Attitude** | | | |
| **Personal Hygiene** |  |  |  |
| I am interested in increasing my knowledge about hygiene measures |  |  | 0.164 |
| Yes | 702 (85.3) | 240 (87.9) |  |
| No | 121 (14.7) | 33 (12.1) |  |
| Wearing a facemask is important during COVID-19 Pandemic |  |  | 0.315 |
| I DO NOT think that wearing a facemask is important | 5 (0.6) | 3 (1.1) |  |
| Because government ordered me to wear a facemask | 18 (2.2) | 1 (0.4) |  |
| Because we can protect our self and others from COVID-19 | 799 (97.1) | 259 (98.5) |  |
| Because my family members asked me to wear a facemask | 1 (0.1) | 0 (0) |  |
| **Personal Hygiene Attitude Score (mean, SD)** | 1.68 (0.62) | 1.64 (0.69) | 0.307 |
| **Social Distancing** |  |  |  |
| Do you think that social distancing measures is important and will help to reduce the spread of COVID-19? |  |  | 0.189 |
| Yes | 805 (97.8) | 271 (99.3) |  |
| No | 18 (2.2) | 2 (0.7) |  |
| I should stay at home when I am not feeling well |  |  | 1.000 |
| Yes | 811 (98.5) | 269 (98.5) |  |
| No | 12 (1.5) | 4 (1.5) |  |
| Which of the following would you consider as main reason for compliance with social distancing measures? |  |  | 0.083 |
| Fear of getting COVID 19 | 433 (52.6) | 152 (55.7) |  |
| Fear of family members getting COVID 19 | 341 (41.4) | 114 (41.8) |  |
| Fear of fines/punitive measures | 49 (6.0) | 7 (2.6) |  |
| Would you willingly participate in the contact tracing app? |  |  | **0.022** |
| Yes | 650 (79.0) | 233 (85.3) |  |
| No | 173 (21.0) | 40 (14.7) |  |
| For how long are you willing to practice social distancing behaviour to keep yourself and others safe? |  |  | 0.152 |
| As long as it takes | 539 (65.5) | 200 (73.3) |  |
| For another 2-3 weeks | 39 (4.7) | 8 (2.9) |  |
| For another 1 month | 64 (7.8) | 17 (6.2) |  |
| For another 3 months | 87 (10.6) | 26 (9.5) |  |
| For another 6 months | 79 (9.6) | 21 (7.7) |  |
| I want social distancing to stop now | 15 (1.8) | 1 (0.4) |  |
| **Social Distancing Attitude Score (mean, SD)** | 3.47 (0.74) | 3.59 (0.62) | **0.014** |
| **Practices** | | | |
| **Social Distancing** |  |  |  |
| How often do you go out of the house in a week (excluding going out for work)? |  |  | 0.140 |
| Never | 67 (8.1) | 29 (10.6) |  |
| 1-2 times | 443 (53.8) | 152 (55.7) |  |
| 3-4 times | 162 (19.7) | 36 (13.2) |  |
| 5-6 times | 89 (10.8) | 34 (12.5) |  |
| More than 7 times | 62 (7.5) | 22 (8.1) |  |
| How many people do you meet face-to-face (<1m) apart everyday (excluding own household)? |  |  | 0.110 |
| 0 | 123 (14.9) | 43 (15.8) |  |
| 1-5 | 273 (33.3) | 106 (38.8) |  |
| 6-10 | 138 (16.8) | 29 (10.6) |  |
| 11-20 | 93 (11.3) | 27 (9.9) |  |
| >20 | 196 (23.8) | 68 (24.9) |  |
| On average, how many places do you go in a day (excluding home)? |  |  | **0.003** |
| 0 | 83 (10.1) | 45 (16.5) |  |
| 1-2 | 629 (76.4) | 199 (72.9) |  |
| 3-4 | 92 (11.2) | 18 (6.6) |  |
| >4 | 19 (2.3) | 11 (4.0) |  |
| **Mental Health** | | | |
| What do you think your probability of getting COVID19 is in the next 1 month? |  |  | **<0.001** |
| 0%, I will not get infected by COVID-19 | 177 (21.5) | 96 (35.2) |  |
| <25% | 433 (52.6) | 122 (44.7) |  |
| <50% | 164 (19.9) | 38 (13.9) |  |
| <75% | 42 (5.1) | 17 (6.2) |  |
| 100% | 7 (0.9) | 0 (0) |  |
| Effects of social distancing on mental health |  |  | **<0.001** |
| Not flourishing | 228 (27.7) | 48 (17.6) |  |
| Flourishing | 595 (72.3) | 225 (82.4) |  |
| Total well-being |  |  | **<0.001** |
| High | 274 (33.3) | 145 (53.1) |  |
| Low | 549 (66.7) | 128 (46.9) |  |
| Emotional well-being |  |  | **<0.001** |
| High | 365 (44.3) | 165 (60.4) |  |
| Low | 458 (55.7) | 108 (39.6) |  |
| Social well-being |  |  | **<0.001** |
| High | 265 (32.2) | 140 (48.7) |  |
| Low | 558 (67.8) | 140 (51.3) |  |
| Psychological well-being |  |  | **<0.001** |
| High | 336 (40.8) | 162 (59.3) |  |
| Low | 487 (59.2) | 111 (40.7) |  |
| **Total well-being Score (mean, SD)** | 44.7 (14.2) | 50.5 (14.5) | **<0.001** |
