# Supplementary material for: Association between well-being and compliance with COVID-19 preventive measures by healthcare professionals: A cross-sectional study
Source: PLoS One. 2021 Jun 7;16(6):e0252835. doi: 10.1371/journal.pone.0252835 (PMC8183980; doi:10.1371/journal.pone.0252835)
Supplement: Attachment — Submitted filename: Response to reviewers.docx [file pone.0252835.s002.docx]

**Response to Reviewers**

Additional Editor Comments:

I have now collected one review from one expert in the field. I was unable to find a second reviewer. However, I am familiar with the topic of this manuscript, therefore I feel confident in making a decision with only one review. The reviewer thinks that the paper can potentially make a valuable contribution, but suggests a major revision. After reading the review and the article, I agree with the reviewer, and therefore I would like to invite you to revise your work for Plos One.

Thank you for your comments and giving us the opportunity to revise our manuscript for publication.

Besides the reviewer's comments, that should all be addressed, I would like to add two more comments from my own reading:

(i) regarding gender differences in compliance to public health recommendations, another paper making this point is: Capraro and Barcelo, 2020;

Capraro, V., & Barcelo, H. (2020). The effect of messaging and gender on intentions to wear a face covering to slow down COVID-19 transmission. Journal of Behavioral Economics for Policy, 4, Special Issue 2, 45-55.

(ii) In the general discussion, you might find useful the perspective article on what social and behavioral science can do to support pandemic response, published by Van Bavel et al. in Nature Human Behaviour.

Van Bavel, J. J., et al. (2020). Using social and behavioural science to support COVID-19 pandemic response. Nature Human Behaviour, 4, 460-471.

Of course, it is not a requirement to cite these papers, I am mentioning them because they look very related to your work and therefore you might find them useful.
I am looking forward for the revision.

Thank you for providing the two references. I have cited (ref 27 and 33) them into my discussion where possible as they support the points raised.

Journal Requirements:

When submitting your revision, we need you to address these additional requirements.

1. Please ensure that your manuscript meets PLOS ONE's style requirements, including those for file naming. The PLOS ONE style templates can be found at

https://journals.plos.org/plosone/s/file?id=wjVg/PLOSOne_formatting_sample_main_body.pdf and

[https://journals.plos.org/plosone/s/file?id=ba62/PLOSOne_formatting_sample_title_authors_affiliations.pdf](about:blank)

Thank you. The manuscript including title page and main text, supporting information have all been modified as per PLOS ONE’s style requirements.

2. Please include additional information regarding the survey or questionnaire used in the study and ensure that you have provided sufficient details that others could replicate the analyses. For instance, if you developed a questionnaire as part of this study and it is not under a copyright more restrictive than CC-BY, please include a copy, in both the original language and English, as Supporting Information.  If the original language is written in non-Latin characters, for example Amharic, Chinese, or Korean, please use a file format that ensures these characters are visible.

S1 Table has been included in the supporting information and it has the original survey questions that were asked for the purpose of this manuscript.

3. Thank you for including your ethics statement:  "No individually identifiable information was obtained during the questionnaire and confidentiality, and anonymity was maintained. Ethics approval was obtained for this study. The NHG DSRB reference number for this study is 2020/00470.".

a.) Please amend your current ethics statement to include the full name of the ethics committee/institutional review board(s) that approved your specific study.
b.) Please provide additional details regarding participant consent. In the ethics statement in the Methods and online submission information, please ensure that you have specified (1) whether consent was informed and (2) what type you obtained (for instance, written or verbal, and if verbal, how it was documented and witnessed). If your study included minors, state whether you obtained consent from parents or guardians. If the need for consent was waived by the ethics committee, please include this information.

If you are reporting a retrospective study of medical records or archived samples, please ensure that you have discussed whether all data were fully anonymized before you accessed them and/or whether the IRB or ethics committee waived the requirement for informed consent. If patients provided informed written consent to have data from their medical records used in research, please include this information.

Once you have amended this/these statement(s) in the Methods section of the manuscript, please add the same text to the “Ethics Statement” field of the submission form (via “Edit Submission”).

For additional information about PLOS ONE ethical requirements for human subjects research, please refer to http://journals.plos.org/plosone/s/submission-guidelines#loc-human-subjects-research.

Thank you. Ethics statement has been modified as per the guidelines and now reads as: “As our study was completely anonymous, it posed no more than minimal risks to respondents and waiver of informed consent would not adversely affect the rights or welfare of study subjects. It qualified for exemption from Singapore’s National Health Group (NHG) Domain Specific Review Board (DSRB) ethics review (2020/00470). It was made clear to participants that by completing the questionnaire, they were giving implied consent for collected information to be used for research purpose.”

4. Please include captions for your Supporting Information files at the end of your manuscript, and update any in-text citations to match accordingly. Please see our Supporting Information guidelines for more information: [http://journals.plos.org/plosone/s/supporting-information](about:blank).

Thank you. Supporting information captions have been added at the end of the manuscript along with updating the in-text citations. Supporting information has also been saved as a separate file to include additional tables and survey.

[Note: HTML markup is below. Please do not edit.]

Reviewers' comments:

Reviewer's Responses to Questions

**Comments to the Author**

1. Is the manuscript technically sound, and do the data support the conclusions?

The manuscript must describe a technically sound piece of scientific research with data that supports the conclusions. Experiments must have been conducted rigorously, with appropriate controls, replication, and sample sizes. The conclusions must be drawn appropriately based on the data presented.

Reviewer #1: Yes

2. Has the statistical analysis been performed appropriately and rigorously?

Reviewer #1: Yes

3. Have the authors made all data underlying the findings in their manuscript fully available?

The [PLOS Data policy](http://www.plosone.org/static/policies.action#sharing) requires authors to make all data underlying the findings described in their manuscript fully available without restriction, with rare exception (please refer to the Data Availability Statement in the manuscript PDF file). The data should be provided as part of the manuscript or its supporting information, or deposited to a public repository. For example, in addition to summary statistics, the data points behind means, medians and variance measures should be available. If there are restrictions on publicly sharing data—e.g. participant privacy or use of data from a third party—those must be specified.

Reviewer #1: Yes

4. Is the manuscript presented in an intelligible fashion and written in standard English?

PLOS ONE does not copyedit accepted manuscripts, so the language in submitted articles must be clear, correct, and unambiguous. Any typographical or grammatical errors should be corrected at revision, so please note any specific errors here.

Reviewer #1: Yes

5. Review Comments to the Author

Please use the space provided to explain your answers to the questions above. You may also include additional comments for the author, including concerns about dual publication, research ethics, or publication ethics. (Please upload your review as an attachment if it exceeds 20,000 characters)

Reviewer #1: This is an interesting and relevant study exploring factors associated with transmission-preventative behaviour among different occupational groups. It is a useful contribution, however I have a number of comments to consider:

Introduction:
1) You could add more about the practical implications of this research on HCPs i.e. why is studying the behaviours of this occupational group so important? Maybe related to maintaining standards of health services? protecting the most vulnerable? role models?

Thank you for your feedback. More literature showing the importance of studying behaviours of HCP and helping to understand the importance of mental health and their association with compliance has been added in the introduction (line 118-121).

2) Related to the above, perhaps clarify if the emphasis is on exploring who and what behaviours need to be targeted to hinder the spread of COVID-19? And/or if the emphasis is on understanding what predicts transmission-preventative behaviour in HCP vs. non-HCP? (this would inform the narrative of the discussion)

Thank you for your feedback. The emphasis is on understanding the differences in KAP and mental health of HCP and non-HCP. Additionally, we also emphasized the importance of well-being and its association with compliance towards preventive behavior among HCP. This has been modified to explain better, ‘The survey also aimed to assess the risk factors associated with compliance towards preventive measures and the role of well-being amongst HCP.’ (line 125-126)

3) Not a lot is said about the wellbeing focus of the survey in the introduction - why is this important to explore?

Thank you for your comment. Importance of well-being has been added in the introduction, ‘

4)Was any particular theory used to inform the focus on KAP?

Thank you for the comment. While we did not replicate any specific model, we adapted the questions based on The Health Belief Model. This has been now added under methods section for better understanding in line 142-143

Method:
1) Were the KAP items adapted from an existing questionnaire or developed by the authors?
Thank you for your comment. The KAP items on personal hygiene and social distancing have been developed by the authors while the questions on mental health have been obtained from the Mental Health Continuum - Short Form (MHC-SF).

This has been added in line 142-143, ‘All questions related to KAP adapted from the Health Belief Model and were developed by the authors of the study’.

2)Perhaps comment on the methodological robustness of the scales e.g. reliability/validity

Thank you for your comment. The lack of validating the survey instrument has been mentioned as a limitation of this study in line 416-417

Results:
1) Did you collect data on the types of HCP within this group? Primary/secondary sector; domiciliary care?

Thank you for your comment. Unfortunately, we did not collect any data on the types of HCP but this is something we can look into in future. This has now been added in the discussion. Line 355-357, ‘Due to limitations of data collection, we were unable to identify the different types of HCP which may help us understand if certain practices, attitudes were more prevalent amongst certain section of healthcare professionals.’

Discussion:
1) Since the discussion focuses on the implications of the research in terms of supporting transmission-preventative behaviour among HCP and non-HCP, maybe the title and aims should be tweaked to reflect this?
Thank you for your comment. While the main outcome of the study is to assess risk factors determining the difference in KAP towards personal hygiene and social distancing measures during COVID-19 amongst HCP and non-HCP (line 65-66), we believe highlighting the key result in the title will be more relevant and provide a succinct outcome of the study.

2) Line 323-324 "However, this needs further research and understanding" - perhaps expand on this, I was not sure what this related to?

Thank you for your comment. This sentence was referring to the previous sentence, ‘*While all respondents avoided standing or sitting close to people, HCP was more sociable and meeting more people, and going to more places in a day, most likely due to their work load and professional demands, making self-isolation difficult*.’

However, this sentence has now been deleted and it has been explained further in line 347 to mention that certain behaviors of HCP need to be understood better and would require further data on the types of HCP, which could throw light on these differences.(line 355-357)

3) Line 340-349: could explore more as to why non-HCP might experience lower sense of wellbeing (furloughed? home-working?)

Thank you for your comment. Higher sense of well-being amongst HCP has been further explained in lines 375-387, ‘However, this is contradictory to higher levels of depression and stress seen amongst HCWs that are assisting COVID-19 patients [33]. A study among nurses showed a negative correlation between perceived stress and happiness scores [13] which can help explain the increased social well-being seen amongst HCP in our study as their professional role may provide them with a large sense of satisfaction and meaning towards protecting the community from COVID-19 even though occupational stress level is likely higher.

A lower sense of well-being seen amongst the non-HCP has also been further explored in line 384-387, ‘A review on impact of COVID-19 on mental health showed student status, unemployment, presence of chronic illness, poor self-rated health were some of the risk factors that predicted stress in the general community [14].’

4) Limitation also of using cross-sectional design

Thank you for your comment. It has been added in line 416-417, ‘Being a cross-sectional study, a causal relationship of risk factors with compliance cannot be established.’

5) The conclusion could build more upon the 'take home' message of the study, in an applied sense i.e. a key practical implication

Thank you for your feedback. We have incorporated the take home message in the conclusion for the abstract as well as the main text.

Abstract, ‘While healthcare professionals were more knowledgeable, had more positive attitudes, their higher sense of total well-being was seen to be more critical to enhance compliance. Therefore, focusing on the well-being of the general population would help to enhance their compliance towards the preventive measures for COVID-19.’ (line 75-78)

Main text, ‘Healthcare professionals were more knowledgeable, showed increased motivation towards practicing personal hygiene and social distancing measures and had better total well-being compared to than non-healthcare professionals. A high level of total well-being may attribute to the high compliance behavior amongst healthcare professionals. Based on the results we believe that by focusing on the total well-being of the general population we can help in increasing their compliance towards various preventive measures.’ (line 421-427)

6. PLOS authors have the option to publish the peer review history of their article ([what does this mean?](https://journals.plos.org/plosone/s/editorial-and-peer-review-process#loc-peer-review-history)). If published, this will include your full peer review and any attached files.


If you choose “no”, your identity will remain anonymous but your review may still be made public.


**Do you want your identity to be public for this peer review?** For information about this choice, including consent withdrawal, please see our [Privacy Policy](https://www.plos.org/privacy-policy).

Reviewer #1: **Yes:** Emma Berry

[NOTE: If reviewer comments were submitted as an attachment file, they will be attached to this email and accessible via the submission site. Please log into your account, locate the manuscript record, and check for the action link "View Attachments". If this link does not appear, there are no attachment files.]

While revising your submission, please upload your figure files to the Preflight Analysis and Conversion Engine (PACE) digital diagnostic tool, <https://pacev2.apexcovantage.com/>. PACE helps ensure that figures meet PLOS requirements. To use PACE, you must first register as a user. Registration is free. Then, login and navigate to the UPLOAD tab, where you will find detailed instructions on how to use the tool. If you encounter any issues or have any questions when using PACE, please email PLOS at [figures@plos.org](mailto:figures@plos.org). Please note that Supporting Information files do not need this step.
